# 3-Methyl-4,5-di­hydro­oxazolium tetra­phenyl­borate

**DOI:** 10.1107/S1600536814003456

**Published:** 2014-02-22

**Authors:** Ioannis Tiritiris, Stefan Saur, Willi Kantlehner

**Affiliations:** a Fakultät Chemie/Organische Chemie, Hochschule Aalen, Beethovenstrasse 1, D-73430 Aalen, Germany

## Abstract

In the cation of the title salt, C_4_H_8_NO^+^·C_24_H_20_B^−^, the C—N bond lengths are 1.272 (2), 1.4557 (19) and 1.4638 (19) Å, indicating double- and single-bond character, respectively. The C—O bond length of 1.3098 (19) Å shows that double-bond character and charge delocalization occurs within the NCO plane of the cation. In the crystal, a C—H⋯π inter­action is present between the methyl­ene H atom of the cation and one phenyl ring of the tetra­phenyl­borate ion. The latter forms an aromatic pocket in which the cation is embedded.

## Related literature   

For the crystal structures of alkali metal tetra­phenyl­borates, see: Behrens *et al.* (2012 ▶). For the synthesis of 1,3-dioxolanes and 1,3-dioxanes from meth­oxy­methyl­ene-*N*,*N*-di­methyl­iminium methyl sulfate, diols and carbonyl compounds, see: Kantlehner & Gutbrod (1979 ▶).
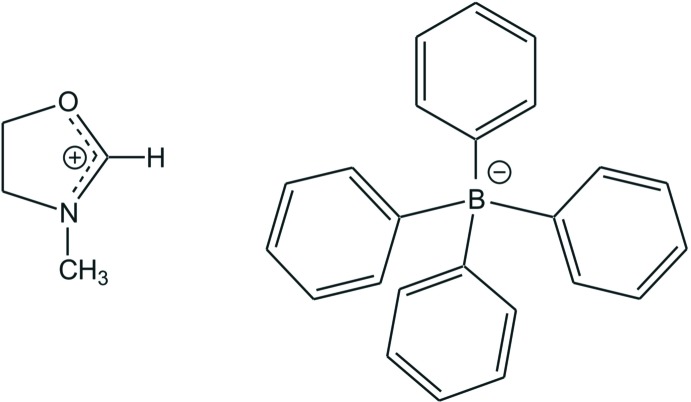


## Experimental   

### 

#### Crystal data  

C_4_H_8_NO^+^·C_24_H_20_B^−^
*M* *_r_* = 405.32
Monoclinic, 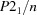

*a* = 11.5335 (5) Å
*b* = 12.7336 (5) Å
*c* = 15.4615 (6) Å
β = 97.209 (2)°
*V* = 2252.77 (16) Å^3^
*Z* = 4
Mo *K*α radiation
μ = 0.07 mm^−1^
*T* = 100 K
0.18 × 0.14 × 0.10 mm

#### Data collection  

Bruker Kappa APEXII DUO diffractometer
38200 measured reflections
5603 independent reflections
4094 reflections with *I* > 2σ(*I*)
*R*_int_ = 0.045

#### Refinement  

*R*[*F* ^2^ > 2σ(*F* ^2^)] = 0.045
*wR*(*F* ^2^) = 0.116
*S* = 1.03
5603 reflections
285 parameters
H atoms treated by a mixture of independent and constrained refinement
Δρ_max_ = 0.48 e Å^−3^
Δρ_min_ = −0.29 e Å^−3^

### 

Data collection: *APEX2* (Bruker, 2008 ▶); cell refinement: *SAINT* (Bruker, 2008 ▶); data reduction: *SAINT*; program(s) used to solve structure: *SHELXS97* (Sheldrick, 2008 ▶); program(s) used to refine structure: *SHELXL97* (Sheldrick, 2008 ▶); molecular graphics: *DIAMOND* (Brandenburg & Putz, 2005 ▶); software used to prepare material for publication: *SHELXL97*.

## Supplementary Material

Crystal structure: contains datablock(s) I, global. DOI: 10.1107/S1600536814003456/kp2465sup1.cif

Structure factors: contains datablock(s) I. DOI: 10.1107/S1600536814003456/kp2465Isup2.hkl

CCDC reference: 987236

Additional supporting information:  crystallographic information; 3D view; checkCIF report

## Figures and Tables

**Table 1:** Hydrogen-bond geometry (Å, °) *Cg*1 is the centroid of the C5–C10 ring.

*D*—H⋯*A*	*D*—H	H⋯*A*	*D*⋯*A*	*D*—H⋯*A*
C2—H2⋯*Cg*1	0.97 (2)	2.31 (2)	3.239 (2)	161 (2)

**Fig. 1.**
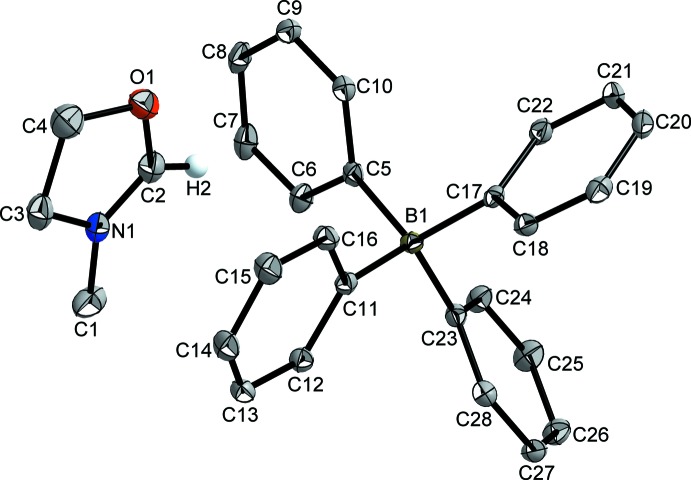
The structure of the title compound with displacement ellipsoids at the 50% probability level. All carbon bonded hydrogen (except of H2) atoms were omitted for the sake of clarity.

**Fig. 2.**
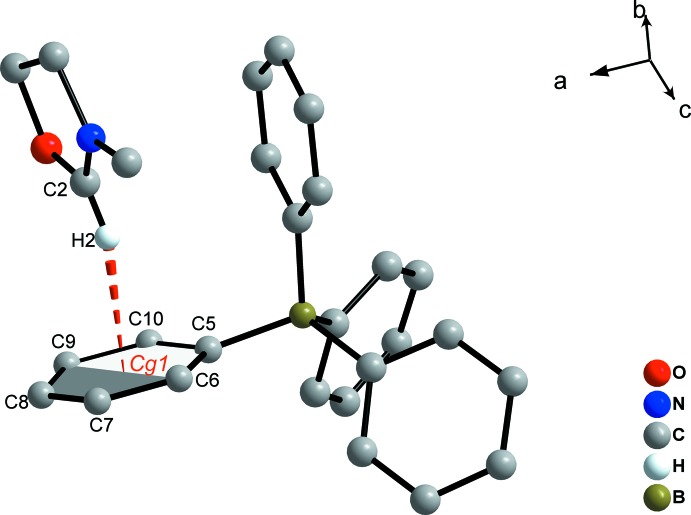
C–H···π interaction (red dashed line) between the hydrogen atom H2 of the cation and the phenyl carbon atoms (centroid) of the tetraphenylborate ion.

## supplementary crystallographic information

### 1. Comment

According to the solved molecular structure of the title salt (Fig. 1) the C1–N1 bond is 1.4557 (19) Å, C3–N1 = 1.4638 (19) Å and C2–N1 = 1.272 (2) Å show single and double bond character, respectively. The C–N1–C angles are: 126.38 (14)° (C1–N1–C2), 122.46 (13)° (C1–N1–C3) and 111.14 (13)° (C3–N1–C2), which indicates a deviation from an ideal trigonal-planar surrounding of the nitrogen centre by the carbon atoms. The C–O length shows with 1.3098 (19) Å double bond character (Fig. 1). The positive charge is delocalized on the plane formed by the atoms N1, C2 and O1. The bond lengths and angles in the tetraphenylborate ion are in good agreement with the data from the crystal structure analysis of the alkali metal tetraphenylborates (Behrens *et al.*, 2012). A C–H···π interaction between the hydrogen atom H2 of the cation and one phenyl ring (centroid) of the tetraphenylborate ion is present (Fig. 2). Here, a short aromatic hydrogen bond (H2···*Cg1* = 2.31 Å) have been determined (Tab. 1). The phenyl rings are forming aromatic pockets, in which the cation is embedded.

### 2. Experimental

The title compound was obtained by reacting of equimolar amounts of *N*,*N*-dimethylformamide with dimethyl sulfate at room temperature giving methoxymethylene-*N*,*N*-dimethyliminium methyl sulfate (I). 3-methyl-4,5-dihydro-oxazolium methyl sulfate (II) was obtained by reacting equimolar amounts of (I) with ethane-1,2-diol under reflux (Kantlehner *et al.*, 1979). The methanol formed was distilled off and (II) was obtained in nearly quantitative yield. In an alternative synthesis, an equimolar mixture of formic acid methyl ester and 2-methylaminoethanol was heated to reflux for four hours, giving 4,5-dihydrooxazole as product. Reaction of 4,5-dihydro-oxazole with dimethyl sulfate for eight hours at 313 K, leads to (II). 1.00 g (5.0 mmol) of crude (II) was dissolved in 20 mL acetonitrile and 1.74 g (5.0 mmol) of sodium tetraphenylborate in 20 mL acetonitrile was added. After stirring for one hour at room temperature, the precipitated sodium methyl sulfate was filtered off. The title compound crystallized from a saturated acetonitrile solution after several days at 273 K, forming colourless single crystals suitable for X-ray analysis.

Dimethyl sulfate is carcinogenic, mutagenic and highly poisonous. During the use appropriate precautions must be taken.

### 3. Refinement

The H atom bound to C2 was located in a difference Fourier map and was refined freely [C—H = 0.97 (2) Å]. The hydrogen atoms of the methyl group were allowed to rotate with a fixed angle around the C–N bonds to best fit the experimental electron density, with *U*_iso_(H) set to 1.5*U*_eq_(C) and d(C—H) = 0.98 Å. The remaining H atoms were placed in calculated positions with d(C—H) = 0.99 Å (H atoms in CH_2_ groups) and (C—H) = 0.95 Å (H atoms in aromatic rings). They were included in the refinement in the riding model approximation, with *U*(H) set to 1.2 *U*_eq_(C).

### Crystal data

**Table:**

C_4_H_8_NO^+^·C_24_H_20_B^−^	*F*(000) = 864
*M**_r_* = 405.32	*D*_x_ = 1.195 Mg m^−^^3^
Monoclinic, *P*2_1_/*n*	Mo *K*α radiation, λ = 0.71073 Å
Hall symbol: -P 2yn	Cell parameters from 38200 reflections
*a* = 11.5335 (5) Å	θ = 2.1–28.3°
*b* = 12.7336 (5) Å	µ = 0.07 mm^−^^1^
*c* = 15.4615 (6) Å	*T* = 100 K
β = 97.209 (2)°	Block, colourless
*V* = 2252.77 (16) Å^3^	0.18 × 0.14 × 0.10 mm
*Z* = 4	

### Data collection

**Table:**

Bruker Kappa APEXII DUO diffractometer	4094 reflections with *I* > 2σ(*I*)
Radiation source: fine-focus sealed tube	*R*_int_ = 0.045
Graphite monochromator	θ_max_ = 28.3°, θ_min_ = 2.1°
φ scans, and ω scans	*h* = −15→15
38200 measured reflections	*k* = −16→16
5603 independent reflections	*l* = −20→20

### Refinement

**Table:**

Refinement on *F*^2^	Primary atom site location: structure-invariant direct methods
Least-squares matrix: full	Secondary atom site location: difference Fourier map
*R*[*F*^2^ > 2σ(*F*^2^)] = 0.045	Hydrogen site location: inferred from neighbouring sites
*wR*(*F*^2^) = 0.116	H atoms treated by a mixture of independent and constrained refinement
*S* = 1.03	*w* = 1/[σ^2^(*F*_o_^2^) + (0.0454*P*)^2^ + 1.1695*P*] where *P* = (*F*_o_^2^ + 2*F*_c_^2^)/3
5603 reflections	(Δ/σ)_max_ < 0.001
285 parameters	Δρ_max_ = 0.48 e Å^−^^3^
0 restraints	Δρ_min_ = −0.29 e Å^−^^3^

### Special details

**Table:**

Geometry. All e.s.d.'s (except the e.s.d. in the dihedral angle between two l.s. planes) are estimated using the full covariance matrix. The cell e.s.d.'s are taken into account individually in the estimation of e.s.d.'s in distances, angles and torsion angles; correlations between e.s.d.'s in cell parameters are only used when they are defined by crystal symmetry. An approximate (isotropic) treatment of cell e.s.d.'s is used for estimating e.s.d.'s involving l.s. planes.
Refinement. Refinement of *F*^2^ against ALL reflections. The weighted *R*-factor *wR* and goodness of fit *S* are based on *F*^2^, conventional *R*-factors *R* are based on *F*, with *F* set to zero for negative *F*^2^. The threshold expression of *F*^2^ > σ(*F*^2^) is used only for calculating *R*-factors(gt) *etc*. and is not relevant to the choice of reflections for refinement. *R*-factors based on *F*^2^ are statistically about twice as large as those based on *F*, and *R*- factors based on ALL data will be even larger.

### Fractional atomic coordinates and isotropic or equivalent isotropic displacement parameters (Å^2^)

**Table:**

	*x*	*y*	*z*	*U*_iso_*/*U*_eq_	
O1	0.29582 (10)	0.82850 (9)	0.05090 (7)	0.0279 (3)	
N1	0.19131 (11)	0.72170 (10)	−0.03783 (8)	0.0213 (3)	
C1	0.12968 (15)	0.62651 (13)	−0.06886 (12)	0.0315 (4)	
H1A	0.1308	0.5763	−0.0207	0.047*	
H1B	0.1682	0.5953	−0.1157	0.047*	
H1C	0.0486	0.6438	−0.0910	0.047*	
C2	0.24007 (14)	0.73900 (13)	0.03931 (10)	0.0235 (3)	
H2	0.2373 (14)	0.6909 (14)	0.0874 (11)	0.025 (4)*	
C3	0.20739 (15)	0.81045 (13)	−0.09530 (10)	0.0259 (3)	
H3A	0.1326	0.8469	−0.1140	0.031*	
H3B	0.2430	0.7881	−0.1473	0.031*	
C4	0.29105 (16)	0.87944 (13)	−0.03477 (11)	0.0321 (4)	
H4A	0.3695	0.8815	−0.0545	0.039*	
H4B	0.2608	0.9520	−0.0327	0.039*	
B1	0.03919 (13)	0.66080 (11)	0.24639 (10)	0.0128 (3)	
C5	0.17365 (12)	0.62690 (10)	0.23467 (8)	0.0138 (3)	
C6	0.19991 (13)	0.53557 (11)	0.18986 (9)	0.0176 (3)	
H6A	0.1382	0.4886	0.1702	0.021*	
C7	0.31271 (13)	0.51105 (12)	0.17311 (10)	0.0218 (3)	
H7A	0.3268	0.4483	0.1429	0.026*	
C8	0.40428 (13)	0.57852 (13)	0.20057 (10)	0.0222 (3)	
H8A	0.4816	0.5619	0.1900	0.027*	
C9	0.38210 (13)	0.67054 (12)	0.24360 (9)	0.0209 (3)	
H9A	0.4441	0.7178	0.2619	0.025*	
C10	0.26874 (12)	0.69362 (11)	0.26002 (9)	0.0170 (3)	
H10A	0.2553	0.7570	0.2895	0.020*	
C11	−0.00704 (12)	0.72451 (10)	0.15633 (9)	0.0137 (3)	
C12	−0.07167 (12)	0.67791 (11)	0.08382 (9)	0.0163 (3)	
H12A	−0.0935	0.6063	0.0874	0.020*	
C13	−0.10532 (13)	0.73228 (12)	0.00652 (9)	0.0190 (3)	
H13A	−0.1496	0.6976	−0.0411	0.023*	
C14	−0.07460 (13)	0.83657 (12)	−0.00123 (10)	0.0218 (3)	
H14A	−0.0981	0.8742	−0.0535	0.026*	
C15	−0.00877 (14)	0.88524 (12)	0.06884 (10)	0.0223 (3)	
H15A	0.0136	0.9567	0.0644	0.027*	
C16	0.02451 (13)	0.83001 (11)	0.14535 (9)	0.0177 (3)	
H16A	0.0704	0.8648	0.1921	0.021*	
C17	0.03362 (12)	0.73485 (10)	0.33271 (9)	0.0133 (3)	
C18	−0.04958 (12)	0.81456 (11)	0.33464 (9)	0.0158 (3)	
H18A	−0.1012	0.8287	0.2831	0.019*	
C19	−0.05996 (13)	0.87411 (11)	0.40894 (10)	0.0191 (3)	
H19A	−0.1178	0.9274	0.4074	0.023*	
C20	0.01404 (13)	0.85559 (12)	0.48497 (10)	0.0215 (3)	
H20A	0.0082	0.8964	0.5357	0.026*	
C21	0.09683 (13)	0.77662 (12)	0.48592 (9)	0.0210 (3)	
H21A	0.1481	0.7629	0.5377	0.025*	
C22	0.10522 (12)	0.71728 (11)	0.41142 (9)	0.0173 (3)	
H22A	0.1617	0.6627	0.4140	0.021*	
C23	−0.04396 (12)	0.56074 (10)	0.26411 (9)	0.0140 (3)	
C24	0.00033 (13)	0.46996 (11)	0.30783 (9)	0.0184 (3)	
H24A	0.0827	0.4615	0.3195	0.022*	
C25	−0.07142 (13)	0.39159 (11)	0.33491 (10)	0.0215 (3)	
H25A	−0.0376	0.3313	0.3643	0.026*	
C26	−0.19191 (13)	0.40137 (11)	0.31916 (10)	0.0202 (3)	
H26A	−0.2412	0.3486	0.3380	0.024*	
C27	−0.23932 (12)	0.48955 (11)	0.27534 (9)	0.0170 (3)	
H27A	−0.3217	0.4972	0.2637	0.020*	
C28	−0.16617 (12)	0.56695 (10)	0.24834 (9)	0.0148 (3)	
H28A	−0.2006	0.6263	0.2180	0.018*	

### Atomic displacement parameters (Å^2^)

**Table:**

	*U*^11^	*U*^22^	*U*^33^	*U*^12^	*U*^13^	*U*^23^
O1	0.0312 (6)	0.0292 (6)	0.0229 (6)	−0.0069 (5)	0.0025 (5)	−0.0015 (5)
N1	0.0218 (7)	0.0247 (6)	0.0185 (6)	0.0002 (5)	0.0074 (5)	0.0002 (5)
C1	0.0275 (9)	0.0262 (8)	0.0434 (10)	−0.0083 (7)	0.0149 (8)	−0.0153 (7)
C2	0.0244 (8)	0.0260 (8)	0.0212 (8)	0.0006 (6)	0.0066 (6)	0.0026 (6)
C3	0.0290 (9)	0.0289 (8)	0.0207 (8)	0.0034 (7)	0.0067 (7)	0.0064 (6)
C4	0.0373 (10)	0.0284 (8)	0.0311 (9)	−0.0078 (7)	0.0059 (8)	0.0040 (7)
B1	0.0125 (7)	0.0122 (6)	0.0135 (7)	−0.0008 (5)	0.0010 (6)	0.0005 (6)
C5	0.0154 (7)	0.0155 (6)	0.0103 (6)	0.0013 (5)	0.0013 (5)	0.0044 (5)
C6	0.0193 (7)	0.0156 (6)	0.0186 (7)	0.0010 (5)	0.0050 (6)	0.0036 (5)
C7	0.0256 (8)	0.0207 (7)	0.0208 (8)	0.0073 (6)	0.0097 (6)	0.0050 (6)
C8	0.0159 (7)	0.0335 (8)	0.0183 (7)	0.0081 (6)	0.0059 (6)	0.0078 (6)
C9	0.0143 (7)	0.0324 (8)	0.0159 (7)	−0.0026 (6)	0.0010 (6)	0.0032 (6)
C10	0.0175 (7)	0.0207 (7)	0.0131 (7)	−0.0004 (5)	0.0029 (5)	0.0006 (5)
C11	0.0116 (6)	0.0159 (6)	0.0137 (6)	0.0023 (5)	0.0023 (5)	0.0008 (5)
C12	0.0162 (7)	0.0169 (6)	0.0161 (7)	−0.0001 (5)	0.0033 (5)	−0.0005 (5)
C13	0.0164 (7)	0.0268 (7)	0.0133 (7)	0.0001 (6)	−0.0002 (5)	−0.0014 (6)
C14	0.0230 (8)	0.0267 (8)	0.0153 (7)	0.0030 (6)	0.0002 (6)	0.0075 (6)
C15	0.0286 (8)	0.0187 (7)	0.0194 (7)	−0.0024 (6)	0.0018 (6)	0.0057 (6)
C16	0.0197 (7)	0.0181 (7)	0.0148 (7)	−0.0034 (5)	0.0003 (6)	0.0016 (5)
C17	0.0121 (6)	0.0141 (6)	0.0142 (7)	−0.0029 (5)	0.0035 (5)	0.0021 (5)
C18	0.0138 (7)	0.0164 (6)	0.0172 (7)	−0.0018 (5)	0.0016 (5)	0.0008 (5)
C19	0.0181 (7)	0.0170 (6)	0.0236 (8)	−0.0011 (5)	0.0077 (6)	−0.0020 (6)
C20	0.0244 (8)	0.0252 (7)	0.0168 (7)	−0.0074 (6)	0.0096 (6)	−0.0053 (6)
C21	0.0205 (8)	0.0306 (8)	0.0120 (7)	−0.0060 (6)	0.0023 (6)	0.0024 (6)
C22	0.0160 (7)	0.0207 (7)	0.0159 (7)	0.0003 (5)	0.0041 (6)	0.0037 (6)
C23	0.0163 (7)	0.0137 (6)	0.0121 (6)	−0.0006 (5)	0.0027 (5)	−0.0011 (5)
C24	0.0143 (7)	0.0204 (7)	0.0208 (7)	0.0011 (5)	0.0032 (6)	0.0057 (6)
C25	0.0213 (8)	0.0174 (7)	0.0258 (8)	0.0017 (6)	0.0031 (6)	0.0080 (6)
C26	0.0209 (8)	0.0179 (7)	0.0224 (8)	−0.0056 (6)	0.0055 (6)	0.0011 (6)
C27	0.0136 (7)	0.0188 (7)	0.0186 (7)	−0.0019 (5)	0.0018 (6)	−0.0026 (5)
C28	0.0171 (7)	0.0132 (6)	0.0138 (6)	0.0015 (5)	0.0013 (5)	0.0000 (5)

### Geometric parameters (Å, º)

**Table:**

O1—C2	1.3098 (19)	C12—C13	1.3936 (19)
O1—C4	1.470 (2)	C12—H12A	0.9500
N1—C2	1.272 (2)	C13—C14	1.383 (2)
N1—C1	1.4557 (19)	C13—H13A	0.9500
N1—C3	1.4638 (19)	C14—C15	1.388 (2)
C1—H1A	0.9800	C14—H14A	0.9500
C1—H1B	0.9800	C15—C16	1.388 (2)
C1—H1C	0.9800	C15—H15A	0.9500
C2—H2	0.968 (18)	C16—H16A	0.9500
C3—C4	1.534 (2)	C17—C18	1.3998 (19)
C3—H3A	0.9900	C17—C22	1.4002 (19)
C3—H3B	0.9900	C18—C19	1.394 (2)
C4—H4A	0.9900	C18—H18A	0.9500
C4—H4B	0.9900	C19—C20	1.383 (2)
B1—C23	1.638 (2)	C19—H19A	0.9500
B1—C5	1.642 (2)	C20—C21	1.386 (2)
B1—C11	1.642 (2)	C20—H20A	0.9500
B1—C17	1.642 (2)	C21—C22	1.391 (2)
C5—C10	1.4036 (19)	C21—H21A	0.9500
C5—C6	1.4060 (19)	C22—H22A	0.9500
C6—C7	1.394 (2)	C23—C28	1.4022 (19)
C6—H6A	0.9500	C23—C24	1.4027 (19)
C7—C8	1.386 (2)	C24—C25	1.394 (2)
C7—H7A	0.9500	C24—H24A	0.9500
C8—C9	1.387 (2)	C25—C26	1.386 (2)
C8—H8A	0.9500	C25—H25A	0.9500
C9—C10	1.394 (2)	C26—C27	1.388 (2)
C9—H9A	0.9500	C26—H26A	0.9500
C10—H10A	0.9500	C27—C28	1.3949 (19)
C11—C12	1.3982 (19)	C27—H27A	0.9500
C11—C16	1.4076 (19)	C28—H28A	0.9500
			
C2—O1—C4	107.37 (12)	C13—C12—C11	122.67 (13)
C2—N1—C1	126.38 (14)	C13—C12—H12A	118.7
C2—N1—C3	111.14 (13)	C11—C12—H12A	118.7
C1—N1—C3	122.46 (13)	C14—C13—C12	120.29 (14)
N1—C1—H1A	109.5	C14—C13—H13A	119.9
N1—C1—H1B	109.5	C12—C13—H13A	119.9
H1A—C1—H1B	109.5	C13—C14—C15	118.81 (13)
N1—C1—H1C	109.5	C13—C14—H14A	120.6
H1A—C1—H1C	109.5	C15—C14—H14A	120.6
H1B—C1—H1C	109.5	C16—C15—C14	120.29 (14)
N1—C2—O1	115.50 (14)	C16—C15—H15A	119.9
N1—C2—H2	123.9 (10)	C14—C15—H15A	119.9
O1—C2—H2	120.6 (10)	C15—C16—C11	122.56 (13)
N1—C3—C4	100.93 (12)	C15—C16—H16A	118.7
N1—C3—H3A	111.6	C11—C16—H16A	118.7
C4—C3—H3A	111.6	C18—C17—C22	115.37 (12)
N1—C3—H3B	111.6	C18—C17—B1	122.04 (12)
C4—C3—H3B	111.6	C22—C17—B1	122.40 (12)
H3A—C3—H3B	109.4	C19—C18—C17	122.81 (13)
O1—C4—C3	104.28 (12)	C19—C18—H18A	118.6
O1—C4—H4A	110.9	C17—C18—H18A	118.6
C3—C4—H4A	110.9	C20—C19—C18	119.99 (14)
O1—C4—H4B	110.9	C20—C19—H19A	120.0
C3—C4—H4B	110.9	C18—C19—H19A	120.0
H4A—C4—H4B	108.9	C19—C20—C21	118.92 (13)
C23—B1—C5	113.26 (11)	C19—C20—H20A	120.5
C23—B1—C11	113.04 (11)	C21—C20—H20A	120.5
C5—B1—C11	104.30 (10)	C20—C21—C22	120.33 (14)
C23—B1—C17	103.14 (10)	C20—C21—H21A	119.8
C5—B1—C17	112.00 (11)	C22—C21—H21A	119.8
C11—B1—C17	111.35 (11)	C21—C22—C17	122.56 (14)
C10—C5—C6	115.41 (13)	C21—C22—H22A	118.7
C10—C5—B1	121.73 (12)	C17—C22—H22A	118.7
C6—C5—B1	122.46 (12)	C28—C23—C24	115.32 (12)
C7—C6—C5	122.73 (14)	C28—C23—B1	121.59 (12)
C7—C6—H6A	118.6	C24—C23—B1	122.41 (12)
C5—C6—H6A	118.6	C25—C24—C23	122.73 (13)
C8—C7—C6	119.83 (14)	C25—C24—H24A	118.6
C8—C7—H7A	120.1	C23—C24—H24A	118.6
C6—C7—H7A	120.1	C26—C25—C24	120.19 (13)
C7—C8—C9	119.42 (14)	C26—C25—H25A	119.9
C7—C8—H8A	120.3	C24—C25—H25A	119.9
C9—C8—H8A	120.3	C25—C26—C27	118.91 (13)
C8—C9—C10	119.94 (14)	C25—C26—H26A	120.5
C8—C9—H9A	120.0	C27—C26—H26A	120.5
C10—C9—H9A	120.0	C26—C27—C28	120.12 (13)
C9—C10—C5	122.64 (14)	C26—C27—H27A	119.9
C9—C10—H10A	118.7	C28—C27—H27A	119.9
C5—C10—H10A	118.7	C27—C28—C23	122.72 (13)
C12—C11—C16	115.36 (12)	C27—C28—H28A	118.6
C12—C11—B1	123.65 (12)	C23—C28—H28A	118.6
C16—C11—B1	120.83 (12)		
			
C1—N1—C2—O1	176.49 (14)	C14—C15—C16—C11	−0.8 (2)
C3—N1—C2—O1	−1.79 (19)	C12—C11—C16—C15	1.7 (2)
C4—O1—C2—N1	−4.22 (19)	B1—C11—C16—C15	177.28 (13)
C2—N1—C3—C4	6.54 (17)	C23—B1—C17—C18	−91.56 (14)
C1—N1—C3—C4	−171.83 (14)	C5—B1—C17—C18	146.32 (12)
C2—O1—C4—C3	7.95 (17)	C11—B1—C17—C18	29.96 (17)
N1—C3—C4—O1	−8.35 (16)	C23—B1—C17—C22	83.19 (15)
C23—B1—C5—C10	−148.97 (12)	C5—B1—C17—C22	−38.93 (17)
C11—B1—C5—C10	87.71 (14)	C11—B1—C17—C22	−155.29 (12)
C17—B1—C5—C10	−32.83 (17)	C22—C17—C18—C19	1.08 (19)
C23—B1—C5—C6	38.57 (17)	B1—C17—C18—C19	176.17 (12)
C11—B1—C5—C6	−84.75 (14)	C17—C18—C19—C20	0.1 (2)
C17—B1—C5—C6	154.70 (12)	C18—C19—C20—C21	−0.7 (2)
C10—C5—C6—C7	1.4 (2)	C19—C20—C21—C22	0.1 (2)
B1—C5—C6—C7	174.27 (13)	C20—C21—C22—C17	1.2 (2)
C5—C6—C7—C8	−0.4 (2)	C18—C17—C22—C21	−1.7 (2)
C6—C7—C8—C9	−0.8 (2)	B1—C17—C22—C21	−176.79 (13)
C7—C8—C9—C10	1.0 (2)	C5—B1—C23—C28	−159.23 (12)
C8—C9—C10—C5	0.0 (2)	C11—B1—C23—C28	−40.87 (17)
C6—C5—C10—C9	−1.2 (2)	C17—B1—C23—C28	79.50 (15)
B1—C5—C10—C9	−174.13 (13)	C5—B1—C23—C24	30.67 (18)
C23—B1—C11—C12	−27.84 (18)	C11—B1—C23—C24	149.03 (13)
C5—B1—C11—C12	95.62 (14)	C17—B1—C23—C24	−90.60 (15)
C17—B1—C11—C12	−143.40 (13)	C28—C23—C24—C25	−0.7 (2)
C23—B1—C11—C16	157.00 (12)	B1—C23—C24—C25	169.93 (13)
C5—B1—C11—C16	−79.54 (15)	C23—C24—C25—C26	−0.1 (2)
C17—B1—C11—C16	41.44 (17)	C24—C25—C26—C27	0.7 (2)
C16—C11—C12—C13	−1.5 (2)	C25—C26—C27—C28	−0.4 (2)
B1—C11—C12—C13	−176.90 (13)	C26—C27—C28—C23	−0.5 (2)
C11—C12—C13—C14	0.3 (2)	C24—C23—C28—C27	1.1 (2)
C12—C13—C14—C15	0.7 (2)	B1—C23—C28—C27	−169.69 (13)
C13—C14—C15—C16	−0.5 (2)		

### Hydrogen-bond geometry (Å, º)

Cg1 is the centroid of the C5–C10 ring.

**Table:**

*D*—H···*A*	*D*—H	H···*A*	*D*···*A*	*D*—H···*A*
C2—H2···*Cg*1	0.97 (2)	2.31 (2)	3.239 (2)	161 (2)

## References

[bb1] Behrens, U., Hoffmann, F. & Olbrich, F. (2012). *Organometallics*, **31**, 905–913.

[bb2] Brandenburg, K. & Putz, H. (2005). *DIAMOND* Crystal Impact GbR, Bonn, Germany.

[bb3] Bruker (2008). *APEX2* and *SAINT* Bruker AXS Inc., Madison, Wisconsin, USA.

[bb4] Kantlehner, W. & Gutbrod, H.-D. (1979). *Liebigs Ann. Chem.* pp. 1362–1369.

[bb5] Sheldrick, G. M. (2008). *Acta Cryst.* A**64**, 112–122. 10.1107/S0108767307043930 18156677

